# Reflux esophagitis triggered after *Helicobacter pylori* eradication: a noteworthy demerit of eradication therapy among the Japanese?

**DOI:** 10.3389/fmicb.2015.00566

**Published:** 2015-06-09

**Authors:** Katsunori Iijima, Tomoyuki Koike, Tooru Shimosegawa

**Affiliations:** Division of Gastroenterology, Tohoku University Graduate School of MedicineSendai, Japan

**Keywords:** *H. pylori*, reflux esophagitis, gerd, eradication, gastric acid

## Abstract

In the February 2013 Revision of Insured Medical Treatment, bacterial eradication for all *Helicobacter pylori*-positive individuals in Japan was covered under the insurance scheme. However, reflux esophagitis is believed to occur in approximately 10% of Japanese patients who undergo eradication therapy. Hence, the risk of reflux esophagitis among such cases should be carefully considered, particularly in the treatment for *H. pylori*-positive patients who are otherwise healthy. The eradication of *H. pylori* in cases of *H. pylori*-positive gastritis markedly suppresses gastric inflammation, and inhibits gastric mucosal atrophy and its progression to intestinal metaplasia. In a long-term follow-up study (10–20 years), eradication treatment was found to reduce the risk of subsequent gastric cancer. However, the fact that eradication-induced reflux esophagitis could increase the long-term risk of Barrett's esophagus and esophageal adenocarcinoma should also be considered in the Japanese population. Appropriate treatment with proton pump inhibitors should be taken into consideration for patients undergoing eradication therapy in clinical practice.

## Introduction

Eradication of *Helicobacter pylori* (*H. pylori*) in cases of *H. pylori*-positive gastritis markedly suppresses gastric inflammation, and inhibits gastric mucosal atrophy and its progression to intestinal metaplasia. Therefore, eradication therapy has been found to reduce the risk of subsequent gastric cancer not only in high-risk group of patients such as those who have undergone endoscopic resection for early gastric cancer (Fukase et al., 2008) but also in healthy asymptomatic infected individuals (Fuccio et al., 2009; Ma et al., 2012; Ford et al., 2014). Thus far, two meta-analyses consistently indicated that *H. pylori* eradication significantly reduce the risk of gastric cancer with relative risk (95% CI): 0.65 (0.43–0.98) and 0.66 (0.46–0.95) (Fuccio et al., 2009; Ford et al., 2014), and a single study successfully revealed its significant preventive effect among more than 3000 subjects with long-term follow-up such as 10–20 years with relative risk (95% CI): 0.61 (0.38–0.96) (Ma et al., 2012). Meanwhile, the occurrence of reflux esophagitis after *H. pylori* eradication was first reported in Europe in 1997 (Labenz et al., 1997). Since then, several conflicting results regarding the causal relationship between eradication therapy and reflux esophagitis have been reported. In this article, we describe the association between *H. pylori* infection and reflux esophagitis and review the literature on post-eradication reflux esophagitis.

## Effect of *H. pylori* infection on gastric acid secretion and its association with gastroesophageal reflux disease

Several meta-analyses have confirmed that *H. pylori* infection is inversely correlated with the occurrence of a large spectrum of gastroesophageal reflux diseases (GERDs), ranging from erosive esophagitis and Barrett's esophagus (BE) to esophageal adenocarcinoma (Raghunath et al., 2003; Rokkas et al., 2007; Wang et al., 2009; Fischbach et al., 2012; Xie et al., 2013a,b). This association is more pronounced in Asia, although it has also been observed in Western countries (Raghunath et al., 2003; McColl, 2004; Xie et al., 2013a,b). With regard to the mechanism underlying the inhibition of GERD onset by gastric *H. pylori* infection, the effect of *H. pylori* on gastric acid secretion and the manner in which the infection modifies GERD manifestation through the reflux of gastric acid into the esophagus are important.

The effect of *H. pylori* infection on gastric acid secretion is associated with the spread of infection-associated gastritis (Atherton and Blaser, 2009). In some cases, the region involved in gastric acid secretion in the stomach body is largely unaffected by *H. pylori* infection and the associated gastric inflammation in the gastric antrum; therefore, the secretion of gastric acid is not reduced. In addition, the infection also results in elevated serum gastrin levels. In such cases, the risk of *H. pylori* infection-associated GERD does not decrease, but is, in fact, believed to increase (Iijima et al., 2000). On the other hand, in cases where gastric inflammation caused by *H. pylori* infection affects the entire stomach body (region involved in gastric acid secretion), the suppressive effect induced by inflammatory cells on parietal cells reduces gastric acid secretion. Furthermore, prolonged inflammation of the stomach body results in a decrease in the number of parietal cells owing to gastric mucosal atrophy, thus further reducing acid secretion (Iijima et al., 2004a). Therefore, in cases where *H. pylori* infection suppresses gastric acid secretion, the level of gastric acid produced during gastric reflux decreases; this process is considered to be the main mechanism through which *H. pylori* infection inhibits the onset of GERD (Koike et al., 2001a; Abe et al., 2004; Inomata et al., 2006).

The extent to which *H. pylori* infection reduces acid secretion greatly differs according to the patient's race. Although gastric acid secretion levels were found to remain constant in most patients with *H. pylori* infection from Western countries (Katelaris et al., 1993; Peterson et al., 1993), the majority of Japanese patients with *H. pylori* infection (mostly elderly individuals) exhibited decreased acid secretion (Iijima et al., 2004b, 2014). Thus, host racial differences in the effect of *H. pylori* infection on gastric acid secretion could be responsible for the different degree of inhibitory effect of the infection on the manifestation of GERD between Asian and Western countries.

## Reflux esophagitis after *H. pylori* eradication

Moreover, no consensus has been reached on whether *H. pylori* eradication leads to the onset of reflux esophagitis. There have been some meta-analyses regarding effects of *H. pylori* eradication on the occurrence of GERD (Raghunath et al., 2004; Yaghoobi et al., 2010; Saad et al., 2012; Xie et al., 2013a). Of these, two meta-analyses failed to find any significant association between the two factors (Raghunath et al., 2004; Saad et al., 2012), and one meta-analysis showed that although there was no significant association in overall analysis, *H. pylori* eradication was significantly associated with the risk of subsequently developing GERD among peptic ulcer patients with relative risk (95% CI): 2.0 (1.1–3.9) (Yaghoobi et al., 2010). Another meta-analysis comprising 12 randomized controlled studies showed that *H. pylori* eradication led to a higher risk of GERD with relative risk (95% CI): 2.0 (1.2–3.2) (Xie et al., 2013a). The possible reasons underlying the lack of a consensus include geographic differences and differences between the diseases for which eradication was performed. First, the geographic differences could explain the conflicting results as one meta-analysis clearly indicated that the risk was significant in the subgroup analysis of Asian studies while it was not observed in that of Western studies with relative risk (95% CI): 4.5 (1.7–12.4) vs. 1.2 (0.9–1.6) (Xie et al., 2013a). Yet, other meta-analyses included only a few Asian studies, and they did not perform subgroup analysis in different regional areas (Raghunath et al., 2004; Yaghoobi et al., 2010; Saad et al., 2012). As mentioned previously, this regional difference is attributed to the racial differences in the effect of *H. pylori* infection on gastric acid secretion. In Europeans and Americans, *H. pylori* infection does not lead to a major change in gastric acid secretion, and a minimal increase is observed after eradication (Katelaris et al., 1993; Peterson et al., 1993). In contrast, in Asians, *H. pylori* infection is associated with decreased gastric acid secretion in the majority of the cases (Iijima et al., 2004b, 2014); therefore, eradication is believed to result in the recovery of gastric acid secretion (Koike et al., 2001b).

Furthermore, the wide variance in reports concerning the likelihood of reflux esophagitis after eradication may be explained by the difference in the diseases for which eradication is performed. For example, in cases with *H. pylori*-positive gastric ulcers, reflux esophagitis is known to occur frequently after eradication (Koike et al., 2001b). On the other hand, in cases with duodenal ulcers, the risk of reflux esophagitis is low; in fact, eradication has been reported to improve the previously existing reflux esophagitis in such cases (Ishiki et al., 2004). Therefore, disease-related differences in the acid secretion changes associated with eradication are believed to play a role in the occurrence reflux esophagitis after eradication (Koike et al., 2001a).

On the other hand, increased gastric acid secretion may be a necessary but not a sufficient factor for developing reflux esophagitis after eradication, since a previous study using simultaneous 24 h pH determination in the stomach and esophagus demonstrated that increased gastric acidity after eradication was not accompanied by concomitant increase in gastroesophageal acid reflux (Fukuchi et al., 2005). In addition to increased gastric acid secretion, impairment of anti-reflux mechanism from the stomach into the esophagus such as hiatal hernia may also be important for developing reflux esophagitis after eradication (Hamada et al., 2000; Koike et al., 2001b; Inoue et al., 2004; Kawanishi, 2005). In fact, Koike et al. reported that reflux esophagitis occurred after eradication only in patients with hiatal hernia (Koike et al., 2001b).

A previous study, which examined hospitalized patients with a variety of diseases who underwent eradication in Japan, reported that the frequency of post-eradication reflux esophagitis was 10–18% (Hamada et al., 2000; Koike et al., 2001b; Inoue et al., 2004; Kawanishi, 2005; Take et al., 2009). The post-eradication follow-up examination revealed many cases of patients in whom reflux esophagitis manifested transiently after the eradication and disappeared after several years (Take et al., 2009). In addition, proton pump inhibitors (PPIs) treatment should be effective for curing reflux esophagitis after eradication, although the drugs do not need to be administered to all patients undergoing eradication therapy given that only a sub-group of patients suffer from the disease after eradication. In the future, the course of post-eradication reflux esophagitis (i.e., the percentage of cases in which reflux esophagitis progresses to BE and esophageal adenocarcinoma) should be observed closely with a long-term follow-up (10–20 years).

## Post-eradication reflux esophagitis in healthy asymptomatic *H. pylori*-infected Japanese individuals

Since February 2013, the Japanese national health insurance has covered eradication therapy for all *H. pylori*-positive patients with chronic gastritis, with an aim of reducing the risk of the subsequent development of gastric cancer (Asaka, 2013); hence, eradication therapy is more widely administered at present. Therefore, the estimation of the risk of reflux esophagitis after eradication therapy in *H. pylori*-positive, asymptomatic, healthy individuals is important. A recent large-scale cross-sectional study in healthy Japanese subjects indicated that there was a significant difference in the prevalence of reflux esophagitis between the subjects with chronic *H. pylori* infection and those who underwent successful eradication (2.3 vs. 8.8%) (Minatsuki et al., 2013). However, thus far, the risk of post-eradication reflux esophagitis in the *H. pylori*-positive population has not been assessed in a prospective cohort study in Japan. One report on the efficacy of eradication therapy in 841 *H. pylori*-positive patients in Taiwan, which is in close proximity to Japan, stated that eradication increased the morbidity associated with reflux esophagitis from 13.7 to 27.3% (Lee et al., 2013). In a Korean study involving 421 patients who underwent health check-ups, reflux esophagitis developed in 10.0% of the patients who underwent eradication compared to 4.3% of the patients who did not undergo eradication; thus, the eradication group showed a significantly higher morbidity (Nam et al., 2010). These data indicate that when eradication therapy is administered to the *H. pylori*-positive population in Asia, the morbidity associated with reflux esophagitis is likely to increase in a sub-group of the subjects.

A potential merit of eradication for patients with peptic ulcers, mucosa-associated lymphoid tissue (MALT) lymphoma, or those who undergo endoscopic resection for early gastric cancer, should obviously outweigh the demerits of the therapy including the *de novo* development of reflux esophagitis. Hence, eradication therapy should not be avoided for these patients due to the risk of developing GERD. Even if GERD develops after eradication, PPI treatment should be effective for curing such disorder. However, eradication-induced reflux esophagitis could in theory increase the long-term risk of BE and esophageal adenocarcinoma (Abe et al., 2011). Hence, the preventive effect of *H. pylori* eradication on gastric cancer should be carefully considered at the cost of increasing the risk of reflux esophagitis, in the treatment of healthy asymptomatic individuals with *H. pylori* infection (Blaser, 2010), particularly in Asian populations. Presence of hiatal hernia (Hamada et al., 2000; Koike et al., 2001b; Inoue et al., 2004; Kawanishi, 2005), body gastritis (Hamada et al., 2000; Koike et al., 2001b), and older age (Koike et al., 2001b; Take et al., 2009) are identified as risk factors for reflux esophagitis after *H. pylori* eradication in Japan, hence careful attention needs to be taken in treating such patients.

## Funding

This work was supported in part by a Grant-in-Aid to KI (25460924) from the Ministry of Education, Science, Sports and Culture in Japan.

### Conflict of interest statement

The authors declare that the research was conducted in the absence of any commercial or financial relationships that could be construed as a potential conflict of interest.
